# Maresin 1 Improves Cognitive Decline and Ameliorates Inflammation in a Mouse Model of Alzheimer’s Disease

**DOI:** 10.3389/fncel.2019.00466

**Published:** 2019-10-15

**Authors:** Ping Yin, Xu Wang, Shuang Wang, Yafen Wei, Jiachun Feng, Mingqin Zhu

**Affiliations:** ^1^Department of Neurology and Neuroscience Center, The First Hospital of Jilin University, Changchun, China; ^2^Department of Neurology, Heilongjiang Provincial Hospital, Harbin, China

**Keywords:** Alzheimer’s disease, resolution of inflammation, maresin 1, neuroinflammation, memory

## Abstract

Alzheimer’s disease (AD) is one of the most common neurodegenerative disease. Accumulating evidences suggest an active role of inflammation in the pathogenesis of AD. Inflammation resolution is an active process that terminates inflammation and facilitates the restoration of inflamed tissue to homeostasis. Resolution of inflammation has been shown to be conducted by a group of specialized pro-resolving lipid mediators (SPMs) including lipoxins, resolvins, protectins, and maresins (MaRs). Recent studies have demonstrated that failure of inflammation resolution can lead to chronic inflammation and, hence, contribute to AD progression. We have previously shown that MaR1 can improve neuronal survival and increase microglial phagocytosis of Aβ. However, the effects of MaR1 on animal models of AD have not been reported. In this study, we aim to investigate the effects of MaR1 on behavioral deficits and pathological changes in a mouse model of AD. Mice received bilateral injections of Aβ_42_ protein into the hippocampus, followed by administration of MaR1 by intra-cerebroventricular injection. The behavioral changes in the mice were analyzed using Morris water maze. Immunohistochemistry, Fluoro-Jade B (FJB) staining, cytometric beads array (CBA), and western blot analysis were used to demonstrate molecular changes in the mice hippocampus and cortex. Our results showed that MaR1 treatment significantly improved the cognitive decline, attenuated microglia and astrocyte activation. In addition, we found that MaR1 decreased the pro-inflammatory cytokines TNF-α, IL-6, and MCP-1 production induced by Aβ42 and increased the anti-inflammatory cytokines IL-2, IL-10 secretion with or without Aβ42 stimulation. Moreover, western blot results showed that MaR1 up-regulated the levels of proteins related to survival pathway including PI3K/AKT, ERK and down-regulated the levels of proteins associated with inflammation, autophagy, and apoptosis pathways such as p38, mTOR and caspase 3. To conclude, MaR1 improved the cognitive decline, ameliorated pro-inflammatory glia cells activation via improving survival, enhancing autophagy, inhibiting inflammation and apoptosis pathways. In conclusion, this study shows that inflammation resolution may be a potential therapeutic target for AD.

## Introduction

Alzheimer’s disease (AD) is the most common cause of dementia, with no existing treatment that can significantly delay or reverse AD-associated cognitive decline or pathological changes. The gross histopathology of AD is characterized by brain atrophy, deepening of the cerebral grooves and the enlargement of the cerebral ventricles. Histologically, AD is characterized by extracellular deposition of amyloid-β (Aβ), intracellular accumulation of neurofibrillary tangles (NFTs) (Alzheimer et al., 1995), and chronic inflammation (McGeer et al., 1987, 1988; Griffin et al., 1989; Cacabelos et al., 1994). Aβ has been considered as a main culprit of AD, and the Aβ hypothesis has been dominant for explaining the pathogenesis of AD (Hardy, 2009). There is a plethora of evidence that support the Aβ hypothesis: patients with an extra copy of chromosome 21, where the APP gene locates, develop dementia at an early age; APP transgenic mice exhibit significant cognitive impairment; the toxicity of Aβ has been documented extensively in *in vitro* studies (Del Bo et al., 1995; Combs et al., 2001; Wicklund et al., 2010). Results from genetic studies have shown an association of inflammation-related genes with AD (Griciuc et al., 2013). Further, microglia activation as well as elevated pro-inflammatory mediators observed in postmortem AD brains and in AD mice models support that chronic inflammation is an integral part of AD pathogenesis (Heppner et al., 2015). Inflammation resolution is an active regulatory process in the end stage of inflammatory reaction that can terminate inflammation and initiate repair of damaged tissues rather than passive disappearance of inflammatory mediators as previously believed (Serhan, 2017). Inflammation resolution is mediated by a group of lipid mediators called specialized pro-resolving lipid mediators (SPMs) including lipoxins (LXs), resolvins (Rvs), protectins (NPDs), and maresins (MaRs), all of which are biosynthesized from polyunsaturated fatty acids (PUFAs) via cyclooxygenases (COXs) and lipoxygenases (LOXs) (Serhan, 2014). In humans, studies have found that reduced SPMs lead to failure of inflammation resolution that can contribute to chronic inflammation diseases such as atherosclerosis (Fredman et al., 2016), dry eye pathogenesis (Gao et al., 2015) as well as AD (Wang et al., 2015).

Evidences from recent studies demonstrate that inflammation resolution is impaired in AD and stimulation of inflammation resolution showed beneficial effects in AD related *in vivo* and *in vitro* models (Wang et al., 2015; Zhu et al., 2016). The conversion from dietary FAs to ω-3 FAs, which are precursors of SPMs has been reported to be decreased in the liver of AD patients (Kang and Rivest, 2012). Accordingly, we have previously found that the levels of SPMs were lower in different areas of the postmortem AD brains including the hippocampus and the entorhinal cortex (Lukiw et al., 2005; Wang et al., 2015). Interestingly, results from clinical trials using PUFAs to treat AD patients showed that ω-3 FAs treatment has beneficial effects only on the patients with mild cognitive impairment (MCI) (Yurko-Mauro et al., 2010) but not on late stage AD patients. Therefore, it is plausible that SPMs are the effective factors mediating the protective effects of ω-3 FAs, however, the conversion from FAs to SPMs is decreased in late stage AD patients. Hence, we hypothesized that SPMs treatment is more effective for AD patients. Afterward, we tested this hypothesis on AD related cellular models including neuronal and microglia models and observed that all the types of SPMs could improve neuronal survival, and MaR1 was more effective in microglial phagocytosis of amyloid-β(Aβ)_42_ (Zhu et al., 2016), indicating that inducing inflammation resolution by SPMs especially by MaR1 could be a novel therapeutic strategy for AD. MaR1 synthesis is initiated by the 14-lipoxygenation of DHA to yield 14S-hydro(peroxy)-4Z,7Z,10Z,12E,14S,16Z,19Z-docosahexaenoic acid and then to 13S, 14S-e MaR. This intermediate is then enzymatically hydrolyzed to MaR1 (Deng et al., 2014; Dalli et al., 2016). The biological functions of MaR1 have been showed in various disease models: MaR1 has to stimulate the pro-inflammatory M1 to anti-inflammatory M2 macrophage phenotype shifts and tissue regenerative actions of MaR1 have also been reported (Dalli et al., 2013). Moreover, MaR1 has been reported suppressed oxidative stress in a left pulmonary hilum I/R mouse model (Sun et al., 2017).

However, the effects of MaR1 on AD animal models have not been studied, and the mechanisms underlying the protective effects of MaR1 remain less understood. The aim of this study was to investigate the effects of MaR1 on behavioral deficits and pathological changes induced by intra-hippocampal injection of Aβ_42_ protein in a mouse model along with the molecular mechanisms of action of MaR1.

## Materials and Methods

### Animals

Forty C57BL/6 mice (male, 3–4 months old, weight 26–31 g) were obtained from animal experiment center of Jilin University. The animals were housed at controlled room temperature (22 ± 2°C) and humidity (50–60%) under a 12h:12h light-dark cycle. Free access to water and food was provided. All procedures used in the present study followed the “National Institutes of Health Guide for Care and Use of Laboratory Animals” (Publication No. 85–23, revised 1985) and were approved by the Animal Ethics Committee of Jilin University. Efforts were also made to minimize animal suffering and to reduce the number of animals used.

### Aβ42 and MaR1 Preparation

Aβ42 (Abcam, United Kingdom, ab120301) was prepared as a stock solution at a final concentration of 1 mg/ml in sterile 0.1 M phosphate-buffered saline (PBS), and aliquots were stored at −20°C. Aβ42 solution was aggregated by incubation at 37°C for 4 days before use. MaR1 (Cayman Chemical, United States) was dissolved in alcohol at the concentration of 0.1 μg/μl and was further diluted with PBS to 0.01 μg/μl solution before use.

### Primary Culture of Microglia

Mouse brain mixed glial cells were prepared from whole brains of 1–3 day postnatal C57BL6/J mice and dissociated with a mild mechanical trituration. Cells were seeded in the cell culture bottles (75 cm^2^) pre-coated with poly-D-lysine. The culture medium was DMEM-High glucose supplemented with 10% fetal bovine serum, 1% penicillin/streptomycin (all from Life Sciences, United States) and 0.8% insulin. Microglia were further extracted from the mixed glial cell cultures by mild trypsinization digestion method as previously described (Saura et al., 2003).

### HT22 Cell Culture

HT22 mouse hippocampal neuronal cell line were cultured at 37°C in Dulbecco’s modified Eagle’s medium (DMEM) supplied with 10% fetal bovine serum (FBS) and antibiotic solution (100 U/ml penicillin, 0.1 mg/ml streptomycin) in a humidified atmosphere in the presence of 5% CO2 until they reached confluence, when reached 90% confluence, cells were subcultured after treatment with 0.25% trypsin-EDTA mixture.

### Cell Viability Assay

Cell Counting Kit-8 (CCK-8, meilunbio) was used to assess the viability of HT22 neuronal cell and microglia. Cells were seeded in 96-well plates and cultured for 24 h (5000/well). After treatment with different concentrations of MaR1 (0.005 μM MaR1, 0.05 μM MaR1, 0.5 μM MaR1) for 6 h, 10 μL CCK-8 solution were added into each well. The optical density values were measured by microplate reader (Bio-Rad iMark) at 490 nm.

### Stereotaxic Intra-Hippocampal Aβ42 Injection and Drug Treatments

Mice were randomly divided into 4 groups: (1) Vehicle group (*n* = 10): bilateral injections of 1 μl PBS in the hippocampus and intra-cerebroventricular injection of 1 μl solvent of MaR1; (2) Aβ42 group (*n* = 10): bilateral hippocampal injections of 1 μl Aβ42 solution and intra-cerebroventricular injection of 1 μl solvent of MaR1; (3) MaR1 group (*n* = 10): bilateral hippocampal injections of 1 μl PBS and intra-cerebroventricular injection of 1 μl MaR1 solution; and (4) Aβ42 + MaR1 group (*n* = 10): bilateral hippocampal injections of 1 μl Aβ42 solution and intra-cerebroventricular injection of 1 μl MaR1 solution.

Mice were anesthetized with isoflurane and the heads were fixed on a stereo locator. A volume of 1 μl of MaR1 solution or MaR1 solvent was injected into the ventricle of mouse brains at the following stereotaxic coordinates using a microsyringe: anteroposterior, −0.3 mm from bregma; mediolateral, +1.00 mm from midline; and dorsoventral, −2.2 mm from dura. The needle was removed after 20 min using three intermediate steps with a 1 min inter-step delay to minimize backflow.

Then, the same procedure was used for bilateral hippocampal injections of Aβ42. A volume of 1 μl of Aβ42 solution or PBS was injected into each side at the following stereotaxic coordinates: anteroposterior, −2.46 mm from bregma; mediolateral, ±1.5 mm from midline; and dorsoventral, −2.0 mm from dura. The needle was removed after 5 min. Mice were placed on a thermal pad (32–33°C) till they were awake.

### Morris Water Maze (MWM) Test

To assess the memory and spatial learning ability of mice, MWM test was conducted 7 days after Aβ42 and MaR1 administration. The experimental apparatus consisted of a circular tank (diameter = 100 cm, height = 50 cm) that was divided into four quadrants. It was filled with water and was maintained at 22 ± 2°C. A platform (diameter = 9 cm) was placed in the pool approximately 1.0 cm below the surface of the water in one of the four quadrants. On the four sides of MWM apparatus, different shapes of visual cues were placed on the inside wall of the pool in a diagonal pattern. A non-toxic white food additive titanium dioxide was added to the water in the pool to contrast with the mice color. We used tracking software (Viewer 2 Tracking Software, Ji Liang Instruments, China) to monitor and record each trial. The MWM paradigm consisted of four 90 s trials per day for 5 consecutive days followed by the probe test on the sixth day. At the end of each trial, the mice were dried with a dry cloth and returned back to the home cages, which were kept on a thermal pad. The interval between each trial for a mouse was more than 30 min. For each trial, starting position was different and the mice were allowed to swim while being tracked by the software. The trial ended when the animal reached the platform or when 90 s had elapsed. If the mouse did not reach the platform, it was directed and placed onto the platform for 10 s. A 90 s probe trial was performed on the sixth day to determine memory retention. For this single trial, the submerged platform was removed and each mouse was placed into the quadrant opposite to the quadrant that formerly contained the platform. The number of platform crossing, the time spent in the quadrant containing the platform previously, and escape latency were recorded.

### Immunohistochemistry

Mice were deeply anesthetized with isoflurane and were sacrificed 1 day after they took WMW test. The brains were harvested, fixed in paraformaldehyde, and then saturated with increasing sucrose concentrations (10%, 20%, 30%) in PBS. Brains were frozen and sectioned coronally at 10 μm thickness using a microtome. Fixed sections were incubated at room temperature for 1 h in 5% normal goat serum in PBS, followed by overnight incubation at 4°C with Iba-1 antibody (1:100; Abcam, ab178847) for microglia and GFAP antibody (1:300; Cell Signaling, 3670) for astrocytes. Thereafter, the sections were washed three times with PBS and incubated for 1 h with Goat Anti-Rabbit IgG (1:500; Abcam, ab150080) and Goat Anti-Mouse IgG (1;500, Abcam, ab150113). Sections were then rinsed with PBS three times. For nucleus labeling, the sections were incubated with DAPI (1:1000; Beyotime, C1006). Positive cells were quantified under a Laser scanning confocal microscope in stained sections in the hippocampus and cortex.

ImageJ software (NIH, Bethesda, MD, United States) was used to measure Iba-1 and GFAP immune-positive staining in the CA1 region and cortex. Four sections per brain were analyzed, and multiple images were taken within each section to cover the CA1 region and cortex. Threshold was equally adjusted across all brain sections to highlight the stained area, and particles were analyzed to determine the total stained area. Results were expressed as total area stained per total area analyzed (area fraction).

### Fluoro-Jade B (FJB) Staining

Prior to immunostaining, fixed sections described above were mounted onto slides from distilled water and then air-dried for at least 30 min on a slide warmer at 50°C. Then, the tissue sections were immersed in a basic alcohol solution of 1% sodium hydroxide in 80% ethanol for 5 min. They were then rinsed in 70% ethanol for 2 min, followed by distilled water for 2 min, and then incubated in 0.06% potassium permanganate solution for 10 min. After a water rinse for 1–2 min, the slides were then transferred to a 0.0004% solution of Fluoro-Jade B (EMD Millipore, United States) dissolved in 0.1% acetic acid for 20 min. The slides were then rinsed thrice in distilled water, ensuring that each rinse lasted for 1 min. The slides were dried by paper towel and then air-dried on a slide at 50°C for at least 5 min. The air-dried slides were then immersed in xylene for at least 1 min and coverslipped using DPX mounting medium. FJB-positive cells were quantified under a microscope (Olympus BX51, Japan) in stained sections in the hippocampus and cortex. Four sections per animal were viewed. Results were expressed as the number of FJB positive cells per section.

### Tissue Processing for Western Blot and Cytometric Beads Array (CBA)

For western blot and CBA, anesthetized mice were subjected to cervical dislocation, the hippocampus and cortex were dissected out and immediately frozen in liquid nitrogen. Proteins were extracted in 10X volume/weight with radio-immuno precipitation assay (RIPA) buffer, supplemented with 1% protease inhibitor cocktail and 1% phosphatase inhibitor cocktail (Sigma-Aldrich, United States), and centrifuged at 12,000 rpm at 4°C for 20 min. The supernatant was collected and stored for further analyses.

### Cytometric Beads Array (CBA)

The levels of tumor necrosis factor (TNF)-α, interleukin (IL)-2, IL-4, IL-6, IL-10, Interferon (IFN)-γ and IL-17A in the hippocampus and cortex tissues were analyzed by CBA (BD Biosciences, United States) according to the manufacturer’s manual. Cytokine levels were then quantified using flow cytometry.

### Enzyme-Linked Immunosorbent Assay (ELISA)

The cortex and hippocampus of mice were homogenized with 10X volume/weight normal saline. The levels of monocyte chemoattractant protein (MCP)-1 were measured by ELISA kit (Shanghai Yuan-ye Bioengineering Institute, China) according to the manufacturer’s manual.

### Western Blot

Analysis of proteins by western blot was performed using proteins extracted from the hippocampus. Total protein concentration was determined by a BCA assay kit. Briefly, samples containing 40 μg protein each were mixed with an equal volume of 2X Laemmli sample buffer, and were boiled at 95°C for 5 min. The denatured samples were then loaded on a 10% SDS-PAGE gel, after which the proteins were transferred to a nitrocellulose membrane under 85 mA current overnight at 4°C. The membranes were blocked with 5% non-fatty dry milk at room temperature for 45 min, and then incubated with the following antibodies: anti-actin (1:2500; Abcam, ab8224), anti-LaminA (1:1000; Abcam, ab26300), Anti-GAPDH (1:10000, Abcam, ab181602), anti-phospho-PI3K (1:1000; Abcam, ab182651), anti-PI3K (1:1000; Abcam, ab191606), anti-phospho-AKT (1:5000; Abcam, ab81283), anti-AKT (1:2000; Abcam, ab28422), anti-phospho-p38 (1:1000; Abcam, ab195049), anti-p38 (1:2000; Abcam, ab170099), anti-phospho-ERK1 + ERK2 (1:1000; Abcam, ab201015), anti-ERK1 + ERK2 (1:10000; Abcam, ab184699), anti-caspase 3 (1:5000; Abcam, ab184737), anti-phospho-mTOR (1:1000; Cell signaling, 2971), anti-mTOR (1:1000; Cell signaling, 2972), anti-LC3B (1:3000; Abcam, ab51520), Anti-SQSTM1/p62 (1:500; Abcam, (ab56416) Anti-Beclin 1 (1:2000, Abcam, ab62557), in Tris-buffered saline with 0.1% Tween 20 (TBS-T) at 4°C overnight. After incubation with appropriate secondary antibody, the antigen-antibody complexes were visualized with the ECL chemiluminescence system (Amersham, United Kingdom). The relative densities of bands were analyzed using the ImageJ software.

### Statistical Analysis

All values are expressed as the mean ± SEM. Normal distributions and homogeneity of variance were found for all analyzed categories. All statistical analyses were conducted using GraphPad Prism 7 software (GraphPad Software, La Jolla, CA, United States). Independent *t*-test was used to compare between the two groups, and one-way-ANOVA was used in multi-group pairwise comparison. In all instances, statistical significance was defined as follows: ^∗^*P* < 0.05, ^∗∗^*P* < 0.01, ^∗∗∗^*P* < 0.001.

## Results

### MaR1 Ameliorated Aβ42 Protein-Induced Cognitive Decline

**FIGURE 1:**
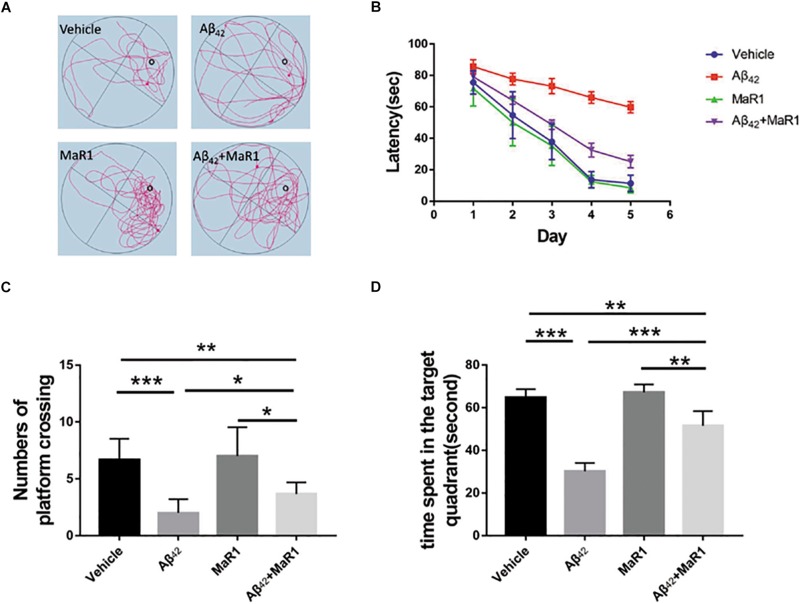
MaR1 improved learning and memory deficits in Aβ_42_ treated mice. **(A)** Typical swimming tracks of the four groups on the sixth day after drug treatment. **(B)** Escape latency gradually decreased with time in all the four groups. On the sixth day after drug treatment, Aβ_42_ treatment group showed significantly increased escape latency compared to Vehicle group, however, Aβ_42_ + MaR1 treatment reversed the changes induced by Aβ_42_ (*P* < 0.05). **(C)** Numbers of platform crossing on the sixth day after drug treatment in probe test. Aβ_42_ treatment significantly decreased the number of platform crossing compared to Vehicle group, while Aβ_42_ + MaR1 treatment attenuated the changes induced by Aβ_42_ (*P* < 0.05). **(D)** Time spent in the target quadrant in the probe test on the sixth day after drug treatment. Aβ_42_ treatment significantly decreased the time spent in the target quadrant compared to Vehicle group, while Aβ_42_ + MaR1 treatment attenuated the changes induced by Aβ_42_ (*P* < 0.001). Data are expressed as mean ± SEM, 10 mice in each group, and statistical significance is defined as follows: ^∗^*P* < 0.05, ^∗∗^*P* < 0.01, ^∗∗∗^*P* < 0.001.

The escape latency for all the groups gradually decreased during 5 consecutive training days, as indicated by Figures 1A,B. Differences gradually became larger in the mean latency between the 4 groups. On the fifth day, the escape latency of Aβ42 group was significantly longer than that of Vehicle group (*P* < 0.05), and the escape latency of Aβ42 + MaR1 group was significantly shorter than that of Aβ42 group (*P* < 0.05). There was no significant difference in escape latency between Vehicle group and MaR1 group. In the probe test, the number of platform crossing and the time spent in the target quadrant by the Aβ42 group were significantly less than those in Vehicle group (*P* < 0.001), and the number of platform crossing and the time spent in the target quadrant by the Aβ42 + MaR1 group were more than those in Aβ42 group (*P* < 0.05). There was no significant difference between Vehicle group and MaR1 group in the platform crossing and the time spent in the target quadrant (Figures 1C,D).

### MaR1 Attenuated Neuronal Degeneration and Glia Activation in the Hippocampus and Cortex of Mice Treated With Aβ42

**FIGURE 2:**
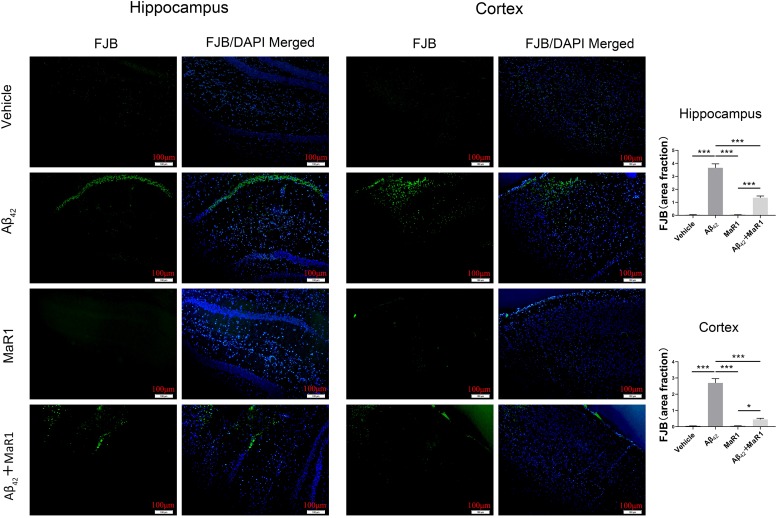
FJB staining for neuronal degeneration in the hippocampus and cortex of AD mouse model. The higher number of degenerative neurons in Aβ_42_ group than that in Vehicle group, and the number of degenerative neurons decreased in Aβ_42_ + MaR1 group than that in Aβ_42_ group (*P* < 0.001). Scale bar = 100 μm, Data are expressed as mean ± SEM, 10 mice in each group, and statistical significance is defined as follows: ^∗^*P* < 0.05, ^∗∗∗^*P* < 0.001.

Neurodegenerative neurons were revealed by FJB staining (Figure 2). In the hippocampus and cortex, the number of degenerative neurons in Aβ42 group was more than that in Vehicle group (*P* < 0.001), while the number of degenerative neurons in Aβ42 + MaR1 group was less than that in Aβ42 group (*P* < 0.001). No significant difference was observed in the number of degenerative neurons between Vehicle group and MaR1 group.

**FIGURE 3:**
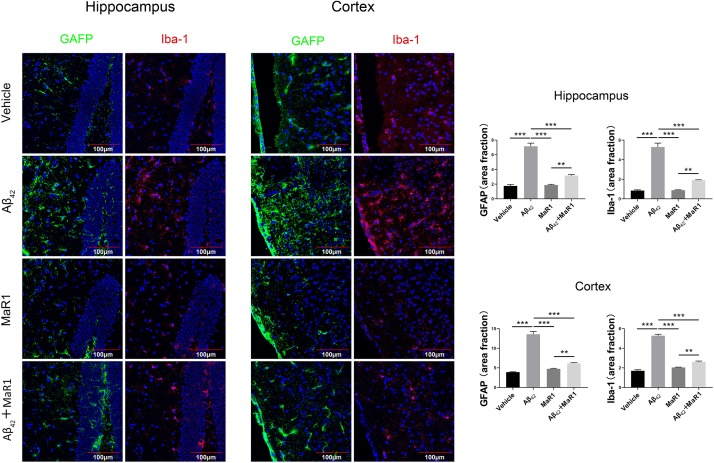
Immunohistochemical staining for microglia and astrocytes in the hippocampus and cortex of AD mouse model. Micrographs and quantification of Iba-1and GFAP positive cells in hippocampus and cortex of the four groups (bar = 100 μm). The percentage of Iba-1 and GFAP positive in Aβ_42_ group was higher than that in Vehicle group, and these changes were attenuated by Aβ_42_ + MaR1 treatment (*P* < 0.001). Data are expressed by mean ± SEM, 10 mice in each group, and statistical significance is defined as follows: ^∗∗^*P* < 0.01, ^∗∗∗^*P* < 0.001.

Iba-1 and GFAP were used to demonstrate microglia and astrocytes, respectively (Figure 3). In the hippocampus and cortex, the percentage of area with Iba-1 and GFAP positive staining cells in Aβ42 group was higher than that in Vehicle group (*P* < 0.001), and the percentage of area with Iba-1 and GFAP positive staining cells in Aβ42 + MaR1 group was lower than that in Aβ42 group (*P* < 0.001). There was no significant difference in the percentage of area with Iba-1 or GFAP positive staining cells between Vehicle group and MaR1 group.

### MaR1 Reduced Pro-inflammatory Cytokine and Chemokine Production and Increased Anti-inflammatory Cytokine Production

**FIGURE 4:**
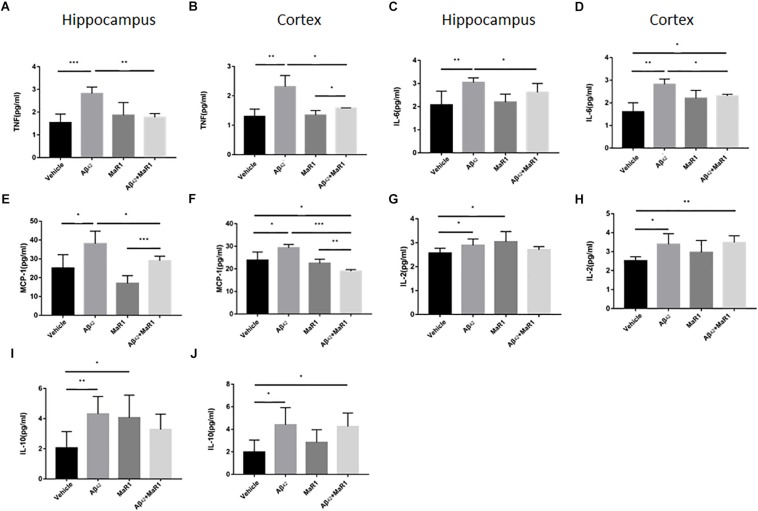
Effects of MaR1 on the secretion of inflammatory cytokines. ELISA and CBA analysis for different inflammatory mediators in the hippocampus and cortex, TNF **(A,B)**, IL-6 **(C,D)**, MCP-1 **(E,F)**, IL-2 **(G,H)**, IL-10 **(I,J)**. **(A–F)** The levels of TNF-α, IL-6 and MCP-1 in Aβ_42_ group were significantly higher than those in Vehicle group (*P* < 0.05), while treatment of Aβ_42_ + MaR1 significantly decreased the levels of TNF-α, IL-6 and MCP-1 induced by Aβ_42_ (*P* < 0.05). **(G–J)** The levels of IL-2 and IL-10 in Aβ_42_ group were significantly higher than those in Vehicle group (*P* < 0.05); MaR1 treatment increased the production of IL-2 and IL-10 in the hippocampus (*P* < 0.05); the levels of IL-2 and IL-10 in Aβ_42_ + MaR1 group were higher than those in Vehicle group in the cortex (*P* < 0.05). Data are expressed as mean ± SEM, 10 mice in each group, and statistical significance is defined as follows: ^∗^*P* < 0.05, ^∗∗^*P* < 0.01, ^∗∗∗^*P* < 0.001.

The production of different cytokines was examined to further investigate the effect of MaR1 on neuroinflammation in AD. The levels of TNF-α, IL-6 and MCP-1 in Aβ42 group were significantly higher than those in Vehicle group (*P* < 0.05), while treatment of Aβ42 + MaR1 significantly decreased the levels of TNF-α, IL-6 and MCP-1 induced by Aβ42 (*P* < 0.05). There was no significant difference in TNF-α, IL-6 and MCP-1 between Vehicle group and MaR1 group (Figures 4A–F). The levels of IL-2 and IL-10 in Aβ42 group were significantly higher than those in Vehicle group in the hippocampus and cortex (*P* < 0.05); MaR1 treatment increased the production of IL-2 and IL-10 in the hippocampus (*P* < 0.05); the levels of IL-2 and IL-10 in Aβ42 + MaR1 group were higher than those in Vehicle group in the cortex but not in the hippocampus (*P* < 0.05) (Figures 4G–J). No difference was observed in the levels of IL-4, IFN-γ and IL-17A among the four groups (data not shown).

### MaR1 Exerted Neuroprotection and Suppressed Neuroinflammation by Modulating Autophagy, Apoptosis, and MAPK Signal Pathways

**FIGURE 5:**
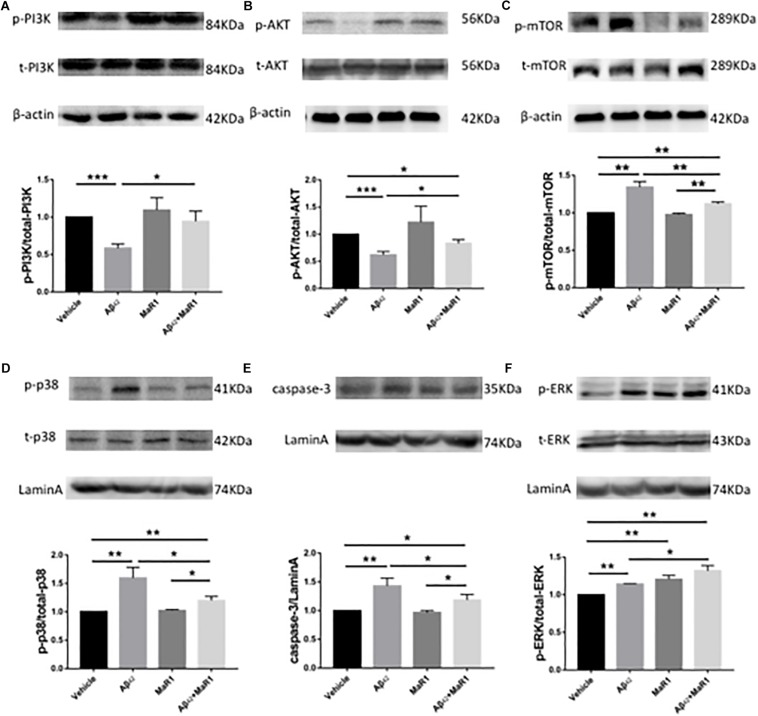
MaR1 altered survival, autophagy, apoptosis, and MAPK signal pathways. Western blots for different proteins and its quantitation in the four groups, PI3K **(A)**, AKT **(B)**, mTOR **(C)**, p38 **(D)**, caspase 3 **(E)**, ERK **(F)**. Compared with Vehicle group, the ratio of p-PI3K/t-PI3K and p-AKT/t-AKT were down-regulated **(A,B)**, while the ratio of p-mTOR/t-mTOR, p-p38/t-p38, as well as the levels of caspase 3 were up-regulated in Aβ_42_ group (*P* < 0.01) **(C–E)**, however, these Aβ_42_ induced changes were reversed by Aβ_42_ + MaR1 treatment. **(F)** The ratio of p-ERK/t-ERK was increased by both, Aβ_42_ (*P* < 0.001) and MaR1 (*P* < 0.01) alone, and was further increased by their co-stimulation compared to Vehicle group (*P* < 0.01) and compared to Aβ_42_ group (*P* < 0.05). Data are expressed as mean ± SEM, 10 mice in each group, and statistical significance is defined as follows: ^∗^*P* < 0.05, ^∗∗^*P* < 0.01, ^∗∗∗^*P* < 0.001.

Compared with Vehicle group, the ratio of p-PI3K/t-PI3K and p-AKT/t-AKT were down-regulated (Figures 5A,B), while the ratio of p-mTOR/t-mTOR, p-p38/t-p38, as well as the levels of caspase 3 were up-regulated in Aβ42 group (*P* < 0.01) (Figures 5C–E), however, these Aβ42 induced changes were reversed by Aβ42 + MaR1 treatment (*P* < 0.05). The ratio of p-ERK/t-ERK was increased by both, Aβ42 (*P* < 0.001) and MaR1 (*P* < 0.01) alone, and was further increased by their co-stimulation as compared to Vehicle group (*P* < 0.01) and Aβ42 group (*P* < 0.05) (Figure 5F).

### MaR1 Exerted Neuroprotection and Suppressed Neuroinflammation by Modulating Autophagy Signal Pathways

**FIGURE 6:**
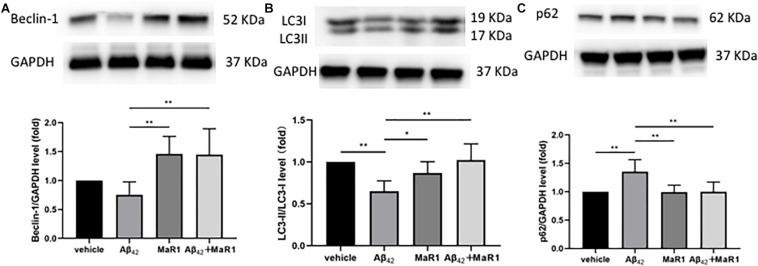
MaR1 altered autophagy signal pathways. Compared with MaR1 group, the level of Beclin-1 **(A)** was decreased in Aβ_42_ group (*P* < 0.01), and increased by Aβ_42_ + MaR1 treatment (*P* < 0.01). Compared with Vehicle and MaR1 group, the ratio of LC3-II/LC3-I **(B)** was down-regulated (*P* < 0.01, *P* < 0.05=, while the levels of p62 **(C)** was up-regulated in Aβ_42_ group (*P* < 0.01), however, these Aβ_42_ induced changes were reversed by Aβ_42_ + MaR1 treatment (*P* < 0.01). Data are expressed as mean ± SEM, 10 mice in each group, and statistical significance is defined as follows: ^∗^*P* < 0.05, ^∗∗^*P* < 0.01.

Compared with Vehicle and MaR1 group, the ratio of LC3-II/LC3-I was down-regulated (*P* < 0.01, *P* < 0.05), while the levels of p62 was up-regulated in Aβ_42_ group (*P* < 0.01), however, these Aβ_42_ induced changes were reversed by Aβ_42_ + MaR1 treatment (*P* < 0.01). Compared with MaR1 group, the level of Beclin-1 was decreased in Aβ_42_ group (*P* < 0.01), and increased by Aβ_42_ + MaR1 treatment (*P* < 0.01) (Figure 6).

### *In vitro* Cytotoxicity Analysis

**FIGURE 7:**
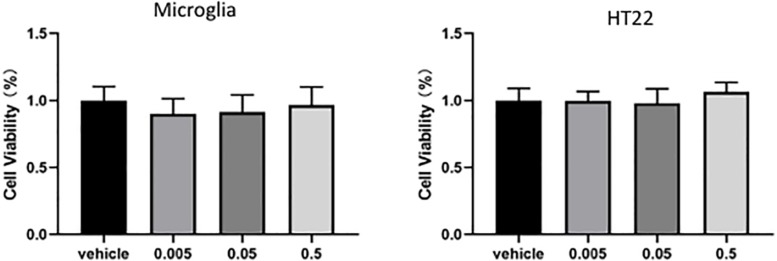
*In vitro* cytotoxicity analysis. There was no significant difference in cell proliferation after treatment with different concentrations MaR1 both in HT22 and in microglia cells.

The cell proliferation rate was at a normal level in HT22 neuronal cell and microglia after MaR1 treatment. There was no significant difference in cell proliferation after treatment with different MaR1 concentrations (Figure 7).

## Discussion

We have previously showed that inflammation resolution is impaired in the AD brains and stimulating resolution of inflammation by SPMs has proved to be beneficial in cellular models of AD, therefore, indicating that resolution of inflammation by SPMs may be a potential therapeutic target for AD (Wang et al., 2015; Zhu et al., 2016). Aspirin-triggered LXA4 (ATL) has been shown to alleviate AD related pathological changes and improve the cognitive impairment in AD animal models (Medeiros et al., 2013; Dunn et al., 2015). However, there is no study regarding the effects of SPMs on *in vivo* AD models. In this study, we have investigated the effects of MaR1 on the behavioral and pathological changes as well as the molecular pathways affected by MaR1 in AD mouse model.

Administrating Aβ_42_ peptide to hippocampus can induce behavioral changes specific to learning and memory (Colaianna et al., 2010; Passos et al., 2010). In the MWM tests, a decrease in the escape latency in all the four groups was observed as the training proceeded, indicating an ongoing spatial learning and memory in the mice. However, the escape latency in Aβ_42_ group was significantly longer compared with Vehicle group on the fifth day, suggesting that mice treated with Aβ_42_ developed learning and memory deficits. But co-stimulation with MaR1 and Aβ_42_ ameliorated the cognitive decline indicated by our observation that the escape latency in Aβ_42_ + MaR1 group was shorter than that in Aβ_42_ group on the fifth day. Similar trends were also found in the probe test, as the number of platform crossings and the time spent in the target quadrant by Aβ_42_ + MaR1 group were longer than those in Aβ_42_ group. All these results suggest that MaR1 could improve behavioral dysfunction induced by Aβ_42_. We also noticed that there were no significant differences in escape latency, cross times, and time spent in target quadrant between Vehicle and MaR1 group. The possible explanation might be that MaR1 alone does not have any effect on normal mice, however, it promotes inflammation resolution only when there are ongoing inflammatory changes.

Consistent with the results of behavioral test, the injection of Aβ_42_ into hippocampus caused neuronal degeneration revealed by FJB staining, which resulted in memory deficits. The number of degenerative neurons in the hippocampus and cortex of Aβ_42_ and MaR1 co-stimulation group was significantly lower than that of Aβ_42_ group, suggesting that MaR1 could restore the behavioral deficits by protecting the neurons from Aβ_42_ toxicity. Increased number of microglia and astrocytes have been observed in postmortem AD brain (Medeiros et al., 2013). Moreover, it has been demonstrated that activation of glia cells including microglia and astrocytes by Aβ leads to elevated production of pro-inflammatory mediators, chronic inflammation, neurodegeneration, decreased glutamate uptake, loss of neuronal synapses, and ultimately cognitive deficits in AD (Prokop et al., 2013; Michaud and Rivest, 2015). In addition, increased cytokines can down-regulate the expression of Aβ phagocytosis receptors on microglia and ultimately result in insufficient microglial phagocytic activity (Hickman et al., 2008). The weakening of Aβ clearance further aggravates the inflammatory response and forms a vicious circle. The immunohistochemical experiments showed that the number of Iba-1 and GFAP positive cells were elevated after Aβ_42_ treatment, however, this trend was reversed by combination treatment of Aβ_42_ and MaR1, suggesting that MaR1 could attenuate inflammation by inhibiting pro-inflammatory activation of microglia and astrocytes. We also observed that the level of MCP-1 was higher in the hippocampus and cortex of Aβ_42_ injected mice, which decreased when mice received combined treatment of Aβ_42_ and MaR1. Therefore, MaR1 may inhibit the chemotaxis of glia by decreasing the secretion of chemokines, which in turn reduces the inflammatory response. Fibrillar Aβ in plaques has been shown to stimulate microglia to secret pro-inflammatory cytokines, including IL-1, IL-6 and TNF-α (Murphy et al., 1998; Combs et al., 2001). There are reports indicating that MaR1 reduces TNF-α and IL-6 secretion in peripheral inflammation disease models such as septic mouse model (Hao et al., 2019), and human periodontal ligament cell model. In line with previous reports, we found MaR1 could reverse the increased levels of TNF-α and IL-6 induced by Aβ_42_ in the central nervous system. The increased levels of IL-6, TNF-α are associated with increased Aβ production, decreased Aβ clearance, tau hyper phosphorylation (Ringheim et al., 1998; Zilka et al., 2012), synaptic dysfunction, and cognitive deficits (Singh-Manoux et al., 2014; Chang et al., 2017). In an aspergillus fumigatus keratitis mouse model, MaR1 was found to increase the expression of anti-inflammatory cytokine IL-10 (Tang et al., 2019). We also observed that both Aβ_42_ and MaR1 could induce the secretion of anti-inflammatory cytokines, IL-2 and IL-10. It has been reported that IL-2 and IL-10 and their signaling pathways are elevated in the brain of AD patients (Strle et al., 2001; Gezen-Ak et al., 2013; Guillot-Sestier et al., 2015a). Some researchers hypothesize that the increase in IL-10 levels may lead to innate immunity repression and microglia dysfunction in Aβ clearance (Guillot-Sestier et al., 2015b), while the others argue that elevated anti-inflammatory cytokines levels is beneficial in decreasing amyloid plaque load, improving synaptic plasticity, and rescuing spine density (Dansokho et al., 2016; Alves et al., 2017). However, it may also be explained by third possibility that Aβ_42_ induces inflammation in the brain, which in turn stimulates IL-10 secretion that plays an immunomodulatory role and facilitates the resolution of inflammatory cascades (Garcia et al., 2017). In summary, MaR1 plays neuroprotective role by down-regulating pro-inflammatory activation of microglia and astrocytes, decreasing pro-inflammatory while increasing anti-inflammatory cytokines production, and promoting resolution of inflammation.

Even though beneficial effects have been reported in a different study using other SPMs in AD mice models (Zhu et al., 2016), there is no research that demonstrates the effects of MaR1 on AD animal models. Moreover, the underlying mechanisms are less understood and the receptor of MaR1 has not been identified. However, in this study, we observed that several signaling pathways involved in inflammation, cellular survival, proliferation, autophagy, apoptosis, and axon formation were altered by MaR1, including PI3K/AKT, ERK1/2, mTOR, p38, and caspase 3.

PI3K/AKT signaling pathway plays an important role in cellular proliferation, growth, survival, motility, and metabolic functions (Kriplani et al., 2015). It has been demonstrated that PI3K/AKT can activate its downstream factors mTOR and Nrf2 (Kim et al., 2013), which are involved in axon branching, autophagy, alleviating oxidative damage, and down-regulating tau protein phosphorylation as well as apoptosis of neurons via inhibiting caspase 3 and glycogen synthase kinase (GSK) -3β (Chami et al., 2016). PI3K/AKT signaling has been found to be impaired in AD patients in previous studies (Wang et al., 2016), Besides AD, the same results have also been observed in other neurodegenerative diseases such as amyotrophic lateral sclerosis (ALS) and Parkinson’s disease (PD) (Recabarren and Alarcon, 2017). All four types of SPMs have been reported to affect the expression of PI3K/AKT pathway and hence, play a protective role: LXA4 protected human or murine macrophages from apoptosis through activation of PI3K/AKT and ERK/Nrf2 pathways (Prieto et al., 2010); RvD1 reduced myocardial infarct size by increasing PI3K/AKT expression (Gilbert et al., 2015); NPD1 induced retinal pigment epithelial cell survival by activating PI3K/AKT signal pathway (Halapin and Bazan, 2010); MaR1 activated PI3K/AKT pathway to down-regulate Nedd4-2 protein and improve Na, K-adenosine triphosphatase (ATPase) activity, and then stimulate alveolar fluid clearance (Zhang et al., 2017). In line with previous findings, we found that PI3K/AKT signaling was down-regulated in Aβ_42_ induced AD mouse model, while MaR1 administration enhanced PI3K/AKT signaling pathway and promoted cell survival, indicating that the protective role of MaR1 is partly due to activation of PI3K/AKT signaling pathway. Furthermore, similar to previous studies in humans and mouse models, we also observed that the mTOR signaling was enhanced in the Aβ_42_ induced AD mouse model. The up-regulation of mTOR could result in reduction of autophagy, which probably is the main cause for abnormal protein aggregation in AD (O’Neill, 2013; Tramutola et al., 2017). This is further supported by studies that show that treatment with mTOR inhibitors improves the cognitive impairment in AD (Tramutola et al., 2017, 2018). Interestingly, previous studies have verified that precursors of SPMs, omega-3 PUFAs, are beneficial for chronic pathological conditions by inhibiting mTOR expression (Kim et al., 2018). In the present study, we observed that MaR1, the downstream product of PUFAs, could also down-regulate the elevated mTOR levels induced by Aβ_42_ treatment, indicating that MaR1 could enhance autophagy by inhibiting mTOR pathway.

The reason for up-regulation of mTOR in AD maybe partly attributed to Ras/ERK, which is an up-stream activating factor for mTOR. Extracellular signal-regulated kinase (ERK) has been found to be elevated in the brain of AD patients (Russo et al., 2002). We observed that ERK signaling pathway was enhanced by both, Aβ_42_ and MaR1 treatment alone or by their combination. Another mitogen-activated protein kinases (MAPKs) family member, p38 signaling pathway, was also enhanced by Aβ_42_ treatment that was reversed by MaR1 treatment. ERK can activate AKT, hence it is the key regulator for neuronal plasticity and survival. In addition, ERK can also activate its downstream effector, cAMP response element binding protein (CREB) (Dineley et al., 2001; Bell et al., 2004), which is involved in synaptic plasticity. However, Aβ-induced activation of ERK pathway in the hippocampus also leads to caspase activation (Chong et al., 2006) and aberrant hyper-phosphorylation of tau protein (Arora et al., 2015). Therefore, subsequent cellular response induced by ERK activation is complicated and can trigger both beneficial and detrimental effects on neuronal cells. The p38 MAPK activation leads to microglia and astrocyte activation and subsequently promotes neuroinflammation in AD (Hensley et al., 1999; Sun et al., 2003; Schnoder et al., 2016). Inhibition of p38 MAPK by p38 MAPK inhibitors can effectively alleviate chronic inflammatory diseases such as rheumatoid arthritis, cardiovascular disease, and inflammatory pain (Cohen et al., 2009; Marber et al., 2011; Arthur and Ley, 2013; Lin et al., 2014), and therefore, may be potential therapies for neurodegenerative diseases including AD (Lee and Kim, 2017). We found p38 signaling was activated in AD by Aβ_42_ and down-regulated after MaR1 treatment, indicating that MaR1 may also play protective role via inhibiting p38 MAPK mediated inflammation.

The down-regulated PI3K/AKT and up-regulated p38 and ERK signaling in AD can induce caspase 3 expression, which is closely related to neuronal apoptosis and is considered as the terminal event preceding cell death. Caspase 3 levels have been demonstrated to be higher in AD brains than in age-matched controls (Shimohama et al., 1999), the same as we have observed in the present study. However, we found that caspase 3 was down-regulated after MaR1 treatment, suggesting that MaR1 plays a protective role by inhibiting apoptosis. To sum up, MaR1 promotes neuronal survival and ameliorates inflammation by up-regulating PI3K/AKT, ERK pathway while down-regulating mTOR, p38, and caspase 3 pathway.

There are some limitations in the current study that can be addressed in further investigations. Firstly, in this study, AD animal model used was made by injecting Aβ_42_ into the hippocampus. Although this method is simple and economical, it could not imitate the pathological changes of AD patients completely, and AD transgenic mice may be a better choice. Secondly, the pathways affected by MaR1 are not only limited to inflammatory pathways, but also other proteins that affect cell survival, axonal growth, and apoptosis, other proteins besides inflammatory cytokines should also be investigated. Thirdly, MaR1 was administered intraventricularly to avoid the restriction of blood-brain barrier (BBB). However, the capacity of MaR1 across the BBB needs to be further studied.

To conclude, resolution of inflammation is impaired in AD, MaR1 could ameliorate memory dysfunction, improve neuronal survival, and reduce secretion of pro-inflammatory while promote anti-inflammatory mediators. MaR1 is beneficial in AD via pathways not only restricted to inflammation, but also through pathways involved in cellular survival, autophagy, axon formation, and apoptosis.

## DATA AVAILABILITY STATEMENT

All datasets generated for this study are included in the manuscript/supplementary files.

## ETHICS STATEMENT

All procedures used in the present study followed the “National Institutes of Health Guide for Care and Use of Laboratory Animals” (Publication No. 85–23, revised 1985) and were approved by the Animal Ethics Committee of Jilin University. Efforts were also made to minimize animal suffering and to reduce the number of animals used.

## Author Contributions

MZ, JF, PY, and XW designed the research. PY, XW, and YW performed the experiments. MZ and PY analyzed the data. MZ, JF, PY, and XW wrote the manuscript. All authors reviewed the manuscript.

## Conflict of Interest

The authors declare that the research was conducted in the absence of any commercial or financial relationships that could be construed as a potential conflict of interest.
